# *Serpentirhabdias orientalis* sp. nov. (nematoda: rhabdiasidae), a new lungworm species in *naja kaouthia* from Thailand: the first record of the genus from the oriental region and an elapid snake

**DOI:** 10.1017/S0031182025000174

**Published:** 2025-03

**Authors:** Vachirapong Charoennitiwat, Supakit Tongpon, Phatthariya Suksuwan, Kittipong Chaisiri, Panithi Laoungbua, Tanapong Tawan, Urusa Thaenkham, Napat Ratnarathorn

**Affiliations:** 1 Department of Helminthology, Faculty of Tropical Medicine, Mahidol University, Bangkok, Thailand; 2 Applied Animal Science Laboratory, Department of Biology, Faculty of Science, Mahidol University, Bangkok, Thailand; 3 Snake Farm, Queen Saovabha Memorial Institute, The Thai Red Cross Society, Bangkok, Thailand

**Keywords:** molecular identification, monocled cobra, morphology, oriental region, *Serpentirhabdias*, snake parasite

## Abstract

A new lungworm species, *Serpentirhabdias orientalis* sp. nov., was discovered in the respiratory organs of the monocled cobra (*Naja kaouthia*) in Thailand, marking the first record of a *Serpentirhabdias* species in both the Oriental Region – Southeast Asia – and in an elapid snake. Morphological analysis revealed distinctive features that clearly differentiate it from all 22 previously known species, such as an elongated body (up to 5858 µm), 6 onchia, a triangular oral opening, and a thin cuticle. The species also exhibited significant differences in several characteristics, such as tail length, distance from the anterior end to the excretory pore and the number of eggs in the uteri, when compared to closely related species such as *Serpentirhabdias moi* and *Serpentirhabdias mussuranae*. Specimens were analysed using light microscopy, and genetic sequencing of *COI* and 28S rRNA confirmed its distinctiveness. Phylogenetic analysis, reinforced by morphological data, revealed a close relationship between *S. orientalis* sp. nov. and Neotropical species, raising intriguing questions about the biogeography and evolutionary history of lungworms. The high prevalence and intensity of infection in the monocled cobras could have ecological consequences, potentially impacting the health of wild snake populations. Increasing wildlife–human interactions highlight the need to monitor parasitic infections for ecological and veterinary insights. These findings contribute to the expanding taxonomy of *Serpentirhabdias* and emphasize the importance of further research into parasitic infections in both wild and captive reptiles, with potential implications for conservation and veterinary management.

## Graphical abstract

**Figure:**
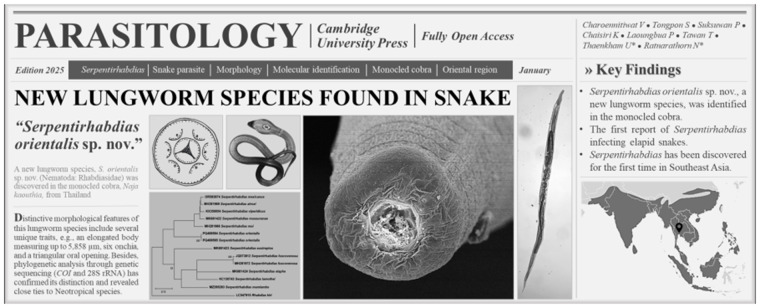

## Introduction

Nematodes of the genus *Serpentirhabdias* (Tkach *et al.*, 2019), within the family Rhabdiasidae (Railliet, 1899), exhibit alternating free-living and parasitic life cycles, infesting the lungs of various snake species. Currently, 22 valid species are described (Kuzmin and Tkach, 2024; Velázquez-Brito *et al*., 2024), characterized by 2–8 mm in length, with rounded anterior and pointed posterior ends. Their mouths feature 6 equally sized lips arranged in pairs, with the presence/absence of onchia in the oesophagostome. This character is useful for dividing *Serpentirhabdias* species into 2 groups: 5 species with onchia and 17 without (Velázquez-Brito *et al*., 2024). Moreover, the 5 species with onchia lack a buccal capsule, while species without onchia usually have a small and thin-walled buccal cavity (Velázquez-Brito *et al*., 2024). The oesophagus is club-shaped with a bulbous posterior. They possess distinct excretory glands and an amphidelphic reproductive system, with seminal receptacles formed by thick-walled parts of the oviduct. The vulva is located at the equatorial or pre-equatorial position, and their tails are conical with pointed tips (Tkach *et al*., 2019).

Species of *Serpentirhabdias* have been documented in various global regions: 10 species in the Neotropics, 7 in the northern part of the Palaearctic, 3 in the Nearctic, 1 in South Africa and 1 in Australia (see Table S1 for species distribution). Notably, *Serpentirhabdias fuscovenosa* (Railliet, 1899; Goodey, 1924) has reportedly been found in both the Nearctic and Palaearctic regions (Baker, 1978, 1987), though they may represent distinct species (Machado *et al*., 2018; Kuzmin *et al*., 2020). Species found in the Palaearctic region, corresponding to reported host distributions (refer to the reptile-database.org – Uetz *et al*., 2024), are typically restricted to the temperate zones of Asia and Europe (Table S1). Importantly, no species have been reported infecting snakes in the Oriental (Southeast Asia) region. Additionally, all *Serpentirhabdias* spp. have been reported infecting snakes primarily in the families Viperidae and Colubridae, with a few species in Dipsadidae and Boidae, but none in Elapidae. This highlights a research gap and lack of attention to the lungworms of snakes within this wildlife, particularly snakes, within this biogeographic region.

The monocled cobra, *Naja kaouthia* Lesson, 1831, is common and widely distributed across the Oriental regions and Thailand (Stuart and Wogan, 2012; Ratnarathorn *et al*., 2019, 2023). Belonging to the family Elapidae, all species of this genus are deadly venomous and of medical importance (Mattison, 2007). This cobra species primarily preys on amphibians, reptiles, small mammals, and chicks (Cox *et al*., 2012), which facilitates its role as a host for parasitic helminths, given its behaviour as an active predator (Jintakune, 2004). Being highly adaptable to anthropogenic environments, this cobra species plays a crucial ecological role within human-altered habitats (Ratnarathorn *et al*., 2024), potentially serving as a reservoir for pathogens and, thereby, presenting an attractive subject for zoonotic research. The study of parasitic infections in snakes highlights their relevance to wildlife health and management, particularly in the context of exotic pet ownership and interactions with wild animals (e.g. Charoennitiwat *et al*., 2024b).

During our survey of parasitic helminths in snakes, a high prevalence of helminths was found inside the lung, trachea and heart of the monocled cobras captured from central Thailand. These helminths were classified as *Serpentirhabdias* due to their general morphology, parasitism in snakes and organ infected (Tkach *et al*., 2019). Only parasitic female nematodes were found in the cobras, as males are reportedly free-living (Tkach *et al*., 2019; Kuzmin *et al*., 2019). Supported by preliminary genetic results, the biological data addressed the species distinction of the specimens in the genus, leading to the suspicion of new species. Consequently, the present study was initiated.

A substantial number of lungworm specimens were analysed using both molecular phylogenetic techniques and detailed morphological examinations. The primary objective of the morphological analysis was to identify distinguishing features of the new nematode species within its taxonomic context, complementing insights from genetic data. Phylogenetic relationships were explored through the reconstruction of genetic sequences, including cytochrome c oxidase subunit 1 (*COI*) and large subunit nuclear ribosomal RNA (28S rRNA). The study aims to provide strong molecular and morphological evidence supporting the description of the newly identified *Serpentirhabdias orientalis* sp. nov., the first report of this species in Southeast Asia and in elapid snakes.

## Materials and methods

### Host and parasite specimen preparation

Five carcasses of wild monocled cobras (*Naja kaouthia*) were obtained from the Snake Farm at Queen Saovabha Memorial Institute, Thai Red Cross Society in Bangkok, Thailand. These cobras, captured by locals and rescuers from the central region, Thailand near Bangkok, died during quarantine. Before dissection, the snakes were examined using criteria from Cox *et al*. (2012), assessing morphological measurements to confirm species and collect host data. Dissections followed protocols by Ratnarathorn and Kongrit (2024) to investigate lung, heart and trachea parasites. Organs were placed in Petri dishes with 0.85% saline and examined under stereomicroscopes (Olympus, SZ30 and SZ51, Japan). Micro-dissecting needles were used to isolate parasites, which were recorded and preserved in 70% ethanol at – 20 °C in the Department of Helminthology, Faculty of Tropical Medicine. Moreover, 10 specimens were preserved in glutaraldehyde for scanning electron microscopy (SEM).

### Morphological study

For morphological studies, 10 complete female helminth specimens were selected from the preserved 70% ethanol stock to create permanent slides. Three specimens in excellent condition were chosen as the holotype and paratypes. Each specimen was stained with acetocarmine and dehydrated by sequential immersion in ethanol (70%, 80%, 90%, 95% and 100%) for 45 min each. For clearing and preparation, specimens were placed in a 1:1 ethanol: xylene solution for 45 min, then briefly in xylene, before being mounted in Permount™ on glass slides, covered with coverslips, and incubated at 60 °C for several days. The remaining specimens (*n* = 47) were mounted using Lactophenol.

**Figure 1.**
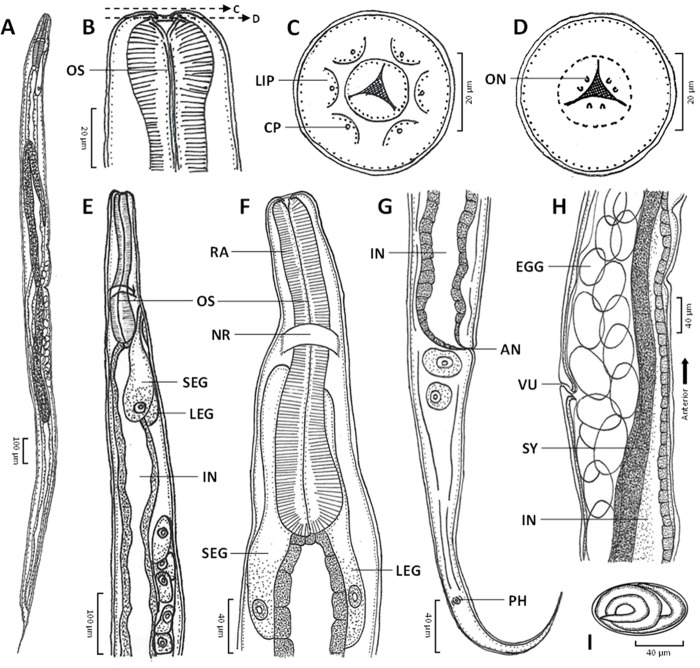
*Serpentirhabdias orientalis* sp. nov. (A) Entire body; (B) anterior end, ventral view, with dashed lines indicating optical cross-sectional views at oesophagostome levels for C and D; (C) anterior end, apical view; (D) optical cross-section at the intermediate-depth level of the oesophagostome, apical view; (E) anterior part of the body, lateral view; (F) anterior part of the body, ventral view; (G) posterior end and tail, lateral view; (H) vulva region at mid-body, lateral view; and (I) egg in the vulva. AN, anus; CP, cephalic papilla; IN, intestine; LEG, large excretory gland; NR, nerve ring; ON, onchia; OS, oesophagus; PH, phasmid; RA, radial muscle of the oesophagus; SEG, small excretory gland; SY, syngonium; and VU, vulva.

A comprehensive examination was conducted using an inverted microscope (Zeiss, Primovert, Germany) equipped with a Zeiss Axiocam and ZEN2 blue edition software. All measurements were recorded in micrometers (µm). Taxonomic keys for identifying *Serpentirhabdias* species and relevant morphological features were derived from Tkach *et al*. (2019), Kuzmin *et al*. (2019, 2020, 2021) and Velázquez-Brito *et al*. (2024). Illustrations (see Figure 1) were generated using a light microscope with a camera lucida (Leitz, Wetzlar, Germany).

**Figure 2.**
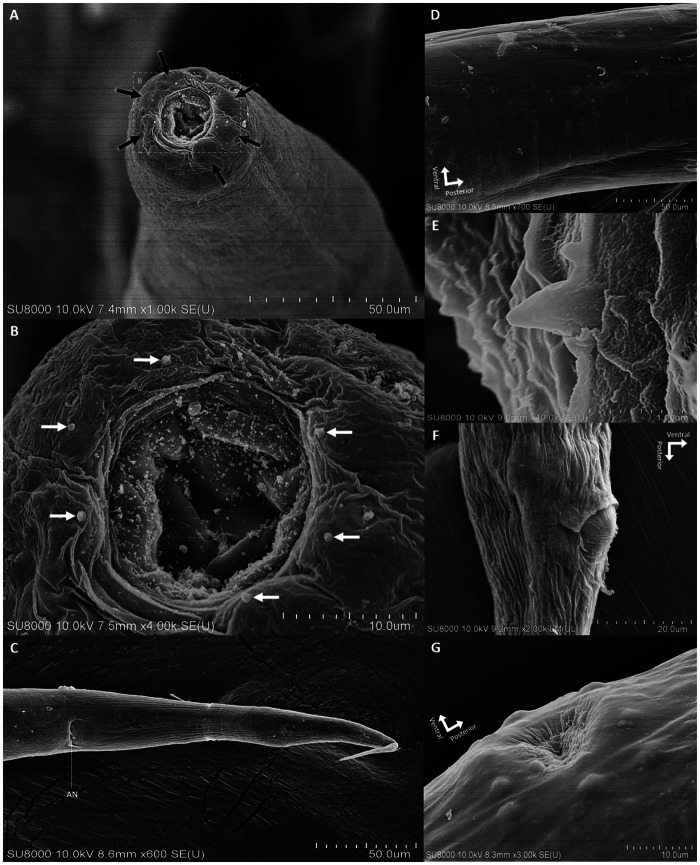
Scanning electron micrographs of *Serpentirhabdias orientalis* sp. nov. (A) anterior region with lips (black arrows), apical view; (B) mouth opening with cephalic papillae (white arrows), apical view, enlarged from A; (C) tail with anus [AN], ventral view; (D) cuticle at mid-body; (E) magnified image of the cephalic papilla; (F) magnified image of the anus, lateral view; and (G) vulva opening, lateral view.

For the SEM analysis, 10 specimens were initially immersed in a solution of 2.5% glutaraldehyde in 0.1 M sucrose phosphate buffer (SPB) for primary fixation, followed by secondary fixation in 1% osmium tetroxide in 0.1 M SPB. The specimens were then dehydrated using ethanol and dried with a critical point dryer (CPD300 auto, Leica, Wetzlar, Germany). They were coated with gold using a sputter coater (Q150R PLUS, Quorum, East Sussex, England). Specimen preparation was carried out at the Department of Tropical Pathology, Faculty of Tropical Medicine, Mahidol University, while SEM imaging (SU8010, Hitachi High-Tech, Japan) was conducted at the Central Instrument Facility, Faculty of Science (Phaya Thai), Mahidol University (see Figure 2 for illustrations).

To assess the morphological variation among the 52 helminth specimens (with 5 incomplete individuals excluded), 19 distinct morphological characteristics were measured, all expressed in the same measurement unit (μm). The measurements are presented as the mean, followed by the standard deviation, with the range in parentheses. These included body length, body width, body width at the oesophago–intestinal junction, buccal cavity width, buccal cavity depth, oesophagus length, oesophageal width at the anterior end, minimum oesophageal width, oesophageal bulb width, the distance from the anterior end to the nerve-ring, the distance from the anterior end to the excretory pore, the distance from the flexure of the posterior syngonium to the tail end, the distance from the anterior end to the vulva, the distance from the anterior end to the flexure of the anterior syngonium, the lengths of the large and small excretory glands, tail length, egg length and egg width (see Tables 1 and S1). A variance–covariance matrix was employed to analyse these 19 characteristics. Additionally, 4 characteristics were included to further examine variations among specimens from different hosts: oesophagus length as a percentage of body length, distance from the anterior end to the vulva as a percentage of body length, tail length, tail length as a percentage of body length and the number of eggs in the uteri (see Table S2). These 4 were analysed in conjunction with the original 19 using a correlation model. The resulting multivariable data matrices were analysed with principal component analysis (PCA) via PAST version 4.06b software (Hammer *et al*., 2001). Both variance–covariance and correlation matrices generated two-dimensional scatter plots that illustrated the morphological variation among specimens and across hosts, along with the corresponding percentage variances. Additionally, to identify the morphological traits causing divergence among host groups, the Discriminant Analysis function in the programme was used.

**Table 1.** Information and measurement characters for *Serpentirhabdias orientalis* sp. nov., *S. viperidicus, S. atroxi, S. mexicanus, S. moi* and *S. mussuranae*. Diagnostic characters for the new species are indicated in bold type. All measurements, in micrometres (μm), are presented as the mean, followed by the standard deviation, with the range in parentheses

Species	*S. orientalis* sp. nov.	*S. viperidicus*	*S. atroxi*	*S. mexicanus*	*S. moi*	*S. mussuranae*
Taxonomic authority	This study	Morais et al., 2017	Kuzmin et al., 2016	Velázquez-Brito et al., 2024	Machado et al., 2018	Kuzmin, Tkach et Melo, 2019
**Host family**	**Elapidae**	**Viperidae**	**Viperidae**	**Viperidae**	**Colubridae**	**Colubridae**
**Host species**	***Naja kaouthia***	***Bothrops moojeni***	***Bothrops atrox***	***Bothrops asper***	***Chironius exoletus***	***Clelia clelia***
**Locality, Region**	**Thailand, Oriental**	**Brazil, Neotropics**	**Brazil, Neotropics**	**Mexico, Neotropics**	**Brazil, Neotropics**	**Brazil, Neotropics**
Infection site(s)	Lung, trachea, & heart	Lung	Lung	Lung	Lung	Lung
No. of parasite specimens	57	34	6	10	10	7
Body length	5,144 ± 431 (3,360–5,858)	5,700 ± 620 (4,350–6,910)	4,100 (3,410–4,540)	5,880 ± 306 (5,247–6,361)	2,500 (2,200–2,600)	4,200 (3,700–4,200)
**Maximum body width**	**207 ± 23 (137–249)**	**384 ± 34 (329–450)**	**234 (168–268)**	**280 ± 42.66 (227–372)**	**109 (103–119)**	**192 (172–225)**
Body width at oesophagus–intestine junction	120 ± 15 (74–148)	229 ± 25 (157–275)	126 (96–157)	171 ± 35.87 (136–236)	–	110 (101–124)
Buccal cavity width	23 ± 3 (14–30)	16 ± 2 (13–22)	12.7 (10–14)	–	10 (10–11)	14 (13–15)
Buccal cavity depth	17 ± 2 (11–24)	18 ± 3 (13 – 23)	11.9 (11–14)	–	7 (6–7)	11 (8–12)
Oesophagus length	351 ± 28 (251–402)	326 ± 20 (288–363)	248 (216–320)	302 ± 19.99 (273–330)	243 (233–256)	284 (274–295)
Oesophagus as % of body length	7 ± 0.4 (6–8)	5.7 ± 0.44 (5–6)	6.1 (5.5–7.8)	5.15 ± 0.47 (4.29–5.73)	9.8 (9.2–10.6)	7.1 (6.7–7.4)
Oesophagus width at anterior end	38 ± 4 (24–51)	62 ± 5 (52–75)	40.5 (32–47)	–	27 (25–29)	36 (34–39)
Oesophagus minimum width	38 ± 4 (24–49)	54 ± 6 (36–63)	34.4 (29–42)	–	–	32 (30–35)
Oesophagus bulb width	71 ± 11 (36–99)	90 ± 10 (56–105)	56.5 (45–69)	75 ± 7.80 (64–86)	42 (40–44)	53 (50–67)
Presence of onchia	Present	Present	Present	Present	Present	Present
Distance from anterior end to nerve-ring	182 ± 23 (111–234)	161 ± 24 (122–210)	133 (112–149)	168 ± 4.94 (159–175)	132 (130–136)	139 (128–157)
**Distance from anterior end to excretory pore**	**279 ± 26 (191–325)**	**217 ± 20 (179–255)**	**175 (139–203)**	**203 ± 7.61 (190–215)**	**140 (136–146)**	**168 (160–185)**
Length of smaller excretory gland	521 ± 44 (340–593)	407 ± 140 (126–662)	400 (309–512)	Only one gland: 429 ± 60 (360–495)	211 (207–219)	401 (334–468)
Length of larger excretory gland	555 ± 46 (362–621)	515 ± 96 (294–660)	423 (344–515)		258 (245–288)	453 (405–489)
Distance from anterior end to vulva	2,387 ± 237 (1,497–2,950)	2,620 ± 250 (2,090–3,190)	1,940 (1,650–2,160)	2,650 ± 230 (2,330–2,990)	1,160 (1,010–1,190)	1,900 (1,800–2,000)
Distance from anterior end to vulva as % of body length	46 ± 3 (42–59)	45 ± 4 (33–52)	47.2 (44.7–49.7)	–	47.0 (44.7–48.3)	48.5 (47.5–49.7)
Distance from anterior end to flexure of anterior syngonium	892 ± 183 (365–1,485)	1585 ± 428 (660–2403)	667 (560–947)	–	409 (356–455)	692 (638–790)
Distance from flexure of posterior syngonium to tail end	986 ± 169 (605–1478)	1218 ± 419 (743–2102)	876 (667–1080)	–	511 (467–580)	865 (784–961)
**Number of eggs in uteri**	**34 ± 16 (12–76)**	**>100**	**>100**	**137 ± 12.58 (120–150)**	**10 (6–13)**	**28 (20–35)**
Egg length	84 ± 7 (60–99)	80 ± 12 (47–106)	72–88	74 ± 6.58 (68–86)	62 (57–71)	83 (70–95)
Egg width	51 ± 6 (30–62)	40 ± 7 (26–60)	39–45	37 ± 2.71 (35–44)	34 (30–39)	38 (33–45)
**Tail length**	**264 ± 24 (209–317)**	**173 ± 23 (123–208)**	**134 (106–160)**	**153 ± 12.75 (122–170)**	**121 (114–128)**	**131 (120–150)**
Tail length as % of body length	5 ± 0.5 (4–7)	3 ± 0.54 (2.1–4.6)	3.3 (2.7–3.7)	2.60 ± 0.24 (2.13–2.99)	4.9 (4.6–5.3)	3.3 (3.1–3.8)

### Molecular and phylogenetic study

For DNA extraction, 2 specimens were homogenized and processed using the DNeasy Blood & Tissue Kit (Qiagen, Germany) according to the manufacturer’s protocol. The extracted genomic DNA was eluted with 30 µL of nuclease-free water.

The amplification focused on partial sequences of 2 genes: the mitochondrial cytochrome c oxidase subunit I (*COI*) and the nuclear 28S ribosomal RNA (28S rRNA), chosen for their utility in molecular identification and genetic diversity studies in nematodes (Chan *et al*., 2020; Thaenkham *et al*., 2022). The primers used were LCO 5′-GGTCAACAAATCATAAAGATATTGGT-3′ and HCO 5′-TAAACTTCAGGGTGACCAAAAAATCA-3′ for *COI*, and #500 5′-ACTTTGAAGAGAGAGTTCAAGAG-3′ and #501 5′-TCGGAAGGAACCAGCTACTA-3′ for 28S rRNA. The expected amplicon lengths were 721 bp for *COI* and 681 bp for 28S rRNA.

PCRs were performed using a T100™ thermocycler (Bio-Rad) in a 30 µL mixture containing 15 µL of 2X i-Taq master mix (Biotechnology, Gyeonggi, South Korea), 10 µM of each primer, and 1 ng/µL of DNA. For the *COI* primers, the thermocycling profile included an initial denaturation at 94 °C for 5 min, followed by 35 cycles of 94 °C for 1 min, 50 °C for 1 min and 72 °C for 1 min, with a final extension at 72 °C for 10 min (Folmer *et al*., 1994). For the 28S rRNA primers, the profile began with an initial denaturation at 94 °C for 3 min, followed by 55 cycles of 94 °C for 30 sec, 54 °C for 30 sec and 72 °C for 1 min, with a final extension at 72 °C for 7 min (Dare *et al.*, 2008). PCR amplicons were visualized on a 1% agarose gel stained with SYBR Safe (Thermo Fisher Scientific, USA). Selected products were sequenced using Fast Next-generation sequencing (Tsingke, Beijing, China). Nucleotide sequences were submitted to the NCBI database with accession numbers PQ469594–95 for *COI* and PQ471682–83 for 28S rRNA.

Partial sequences of the genes were manually inspected and edited using BioEdit version 7.2.5. Phylogenetic analysis was conducted using maximum likelihood in MEGA-X, with sequence alignment performed by ClustalX 2.1. Best-fit nucleotide substitution models were selected for each gene: Tamura-Nei (TN93) with a discrete gamma distribution (+G) and by assuming that a certain fraction of sites is evolutionarily invariable (+I) for *COI* and Kimura 2-parameter (K2) with a discrete gamma distribution (+G) for 28S rRNA. Phylogenetic trees were constructed with 1000 bootstrap replicates to ensure robust analysis (Hall, 1999; Thompson *et al*., 2002; Tamura *et al*., 2013). The *COI* and 28S rRNA analyses incorporated the sequences of all available species within *Serpentirhabdias* and other species of the family Rhabditidae. For the *COI* analysis, *Rhabdias kiri* was used as the outgroup, while various Rhabditid species served as outgroups for the 28S rRNA analysis. All sequences were retrieved from GenBank (see Figure 5).

## Results

### Taxonomy

Phylum: Nematoda Diesing, 1861

Class: Chromadorea Inglis, 1983

Order: Rhabditida Chitwood, 1933

Family: Rhabdiasidae (Railliet, 1899)

Genus: *Serpentirhabdias* (Tkach *et al.*, 2019)

**Figure 3.**
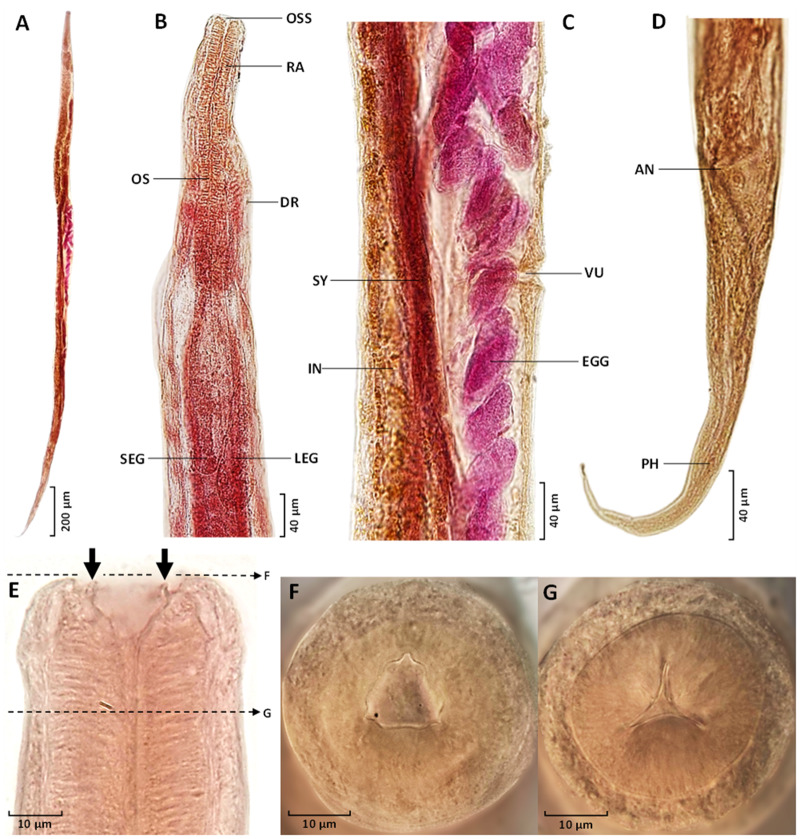
Permanent slides (acetocarmine dye, A–E) and semi-permanent slides (lactophenol, F and G) of *Serpentirhabdias orientalis* sp. nov.: (A) entire body; (B) anterior region, ventral view; (C) vulva region at mid-body, lateral view; (D) posterior end and tail, lateral view; (E) anterior end showing onchia (black arrows), ventral view, with dashed lines indicating optical cross-sectional views at the oral part for F, and at the oesophagus for G; (F) oral part showing lips and triangular opening, apical view; and (G) optical cross-section at the intermediate level of the oesophagus, apical view. A, anus; DR ,deirid; IN, intestine; LEG, large excretory gland; OS, oesophagus; Oss, oesophagostome; PH, phasmid, RA, radial muscle of oesophagus, SEG, small excretory gland; SY, syngonium, and VU, vulva.

Species: *Serpentirhabdias orientalis* sp. nov. (Charoennitiwat *et al.*, 2025; Table 1, Figures 1–3).

**Type-host**: *Naja kaouthia* Lesson, 1831

**Type-locality**: Bangkok’s suburbs (e.g. Don Mueang district, Prawet district, etc.) in Thailand. Coordinates were not recorded.

**Collection date**: November 10^th^, 2020 to August 26^th^, 2023.

**Site of infection**: Lung, trachea and heart

**Parasite intensity**: 6–216 worms, mean approximately 58 (based on 5 host specimens)

**ZooBank LSID**:

**Etymology**: The specific epithet ‘*orientalis*’ indicates that the nematode species is discovered in oriental region. We propose the colloquial English name for this nematode as the ‘Oriental snake lungworm’.

### General description

Based on 57 parasitic adult female specimens (Table 1): body elongated, with anterior end rounded, and posterior end tapering (Figures 1A and 3A). Body length 5144 ± 431 (3360–5858). Width 207 ± 23 (137–249), and width 120 ± 15 (74–148) at oesophago–intestinal junction. Cuticle thin and smooth (Figure 2D). Oral opening triangular and wide in apical view (Figures 1C, 2A, and 3F, G); buccal cavity width 23 ± 3 (14–30) and depth 17 ± 2 (11–24). Oesophagus 351 ± 28 (251–402) in length, representing 7 ± 0.4 (6–8) % of body length, with 38 ± 4 (24–51) width at anterior end and 38 ± 4 (24–49) minimum width; oesophageal bulb 71 ± 11 (36–99) width. Six lips similar in shape and size, arranged in 2 lateral groups of 3 (Figures 1C and 2A). Each lip with small, cone-like papillae (Figure 2B and E). Six onchia project outward from top of radial muscle in oesophagostome (Figures 1B, D, and 3E). Distance from anterior end to nerve-ring 182 ± 23 (111–234); anterior end to excretory pore 279 ± 26 (191–325). Excretory gland length 521 ± 44 (340–593) and 555 ± 46 (362–621) in smaller and larger glands, respectively (Figures 1E, F, and 3B). Distance from anterior end to anterior syngonium flexure 892 ± 183 (365–1485) and from posterior syngonium flexure to tail end 986 ± 169 (605–1478). Distance from anterior end to vulva 2387 ± 237 (1497–2950), representing 46 ± 3 (42–59) % of body length. Uteri containing 34 ± 16 (12–76) eggs (Figures 1H and 3C). Egg size (width × length) 51 ± 6 (30–62) × 84 ± 7 (60–99) (Figure 1I). Tail narrow, elongated, gradually tapering posteriorly (Figures 1G, 2C, and 3D). Tail length 264 ± 24 (209–317), representing 5 ± 0.5 (4–7) % of body length.

### Type materials

A parasitic gravid female specimen, designated as the holotype (Voucher no.: MUMNH-NEM0033; specimen code: SN063_Holotype), and 2 parasitic female paratypes (Voucher nos.: MUMNH-NEM0034–0035; specimen codes: SN127_Paratype1–2) were deposited at the Mahidol University Museum of Natural History. All specimens were collected from the lungs of the monocled cobras (*Naja kaouthia*), with the holotype (project ID: SN063; AASL ID: AAS107 [EL-Nk-029]) collected on August 3^rd^, 2023, and the paratypes (project ID: SN127; AASL ID: AAS091 [EL-Nk-027]) on November 9^th^, 2023, by Vachirapong Charoennitiwat and his team at the Department of Helminthology, Faculty of Tropical Medicine, Mahidol University. Measurements for both the holotype and paratypes are provided in Table S2, with their morphological characteristics consistent with the general description.

### Remarks

*Serpentirhabdias orientalis* sp. nov. is placed within the genus *Serpentirhabdias* based on key features, including 6 laterally arranged lips, a thin body cuticle without noticeable cuticular inflations, a vulva positioned near the mid-body with surrounding papillae, and the absence of a buccal capsule (Figures 2A–G and 3A–G). This species is also characterized by its lung parasitism in snakes, aligning it with other members of the genus (Tkach *et al*., 2019; Kuzmin *et al*., 2016; Velázquez-Brito *et al*., 2024).

The genus *Serpentirhabdias* consists of 22 globally distributed species (Table S1), divided into 2 morphological groups based on the presence or absence of onchia in the oesophagostome. Six species, including *Serpentirhabdias orientalis* sp. nov., possess onchia (Figures 1B, D, and 3E), grouping it with *Serpentirhabdias atroxi* (Kuzmin *et al.*, 2016), *Serpentirhabdias viperidicus* (Morais *et al.*, 2017), *Serpentirhabdias moi* (Machado *et al.*, 2018), *Serpentirhabdias mussuranae* (Kuzmin *et al*., 2019) and *Serpentirhabdias mexicanus* (Velázquez-Brito *et al.*, 2024). All these species share the absence of a buccal capsule. Moreover, these 6 species are categorized into 2 subgroups based on the shape of their oral openings (Velázquez-Brito *et al*., 2024): the rounded-mouth species – *S. atroxi, S. viperidicus* and *S. mexicanus* – and the triangular-mouth species – *S. moi, S. mussuranae* and *S. orientalis* sp. nov (Figures 1C, 2A, and 3F).

*Serpentirhabdias orientalis* sp. nov. exhibits significant morphological distinctions from *S. moi* in several key traits. In particular, *S. orientalis* sp. nov. is much larger than *S. moi* in body length (ave. 5,144 [3360–5858] vs 2500 [2200–2600]), body width (ave. 207 [137–249] vs 109 [103–119]) and tail length (ave. 264 [209–317] vs 121 [114–128]). Further notable differences are observed in the dimensions of the buccal cavity, with *S. orientalis* sp. nov. having both a wider (ave. 23 [14–30]) and deeper (ave. 17 [11–24]) buccal cavity compared to *S. moi* (ave. 10 [10–11] and 7 [6–7], respectively). The length of the oesophagus also varies significantly, as *S. orientalis* sp. nov. has an average length of 351 (251–402), while *S. moi* averages 243 (233–256). Reproductive traits also differ significantly between the 2 species, with *S. orientalis* sp. nov. having a higher average number of eggs in its uteri (ave. 34 [12–76]) compared to *S. moi* (ave. 10 [6–13]). In terms of geographical distribution and host specificity, *Serpentirhabdias orientalis* sp. nov infects the lung, bronchi and heart of the monocled cobra, *Naja kaouthia* (Elapidae) in Southeast Asia, primarily Thailand. In contrast, *S. moi* parasitizes only the lung of the Linnaeus’ Sipo, *Chironius exoletus* (Colubridae), in the Neotropical region, specifically Brazil (Machado *et al*., 2018).

*Serpentirhabdias orientalis* sp. nov. can be differentiated from *S. mussuranae* by its longer tail length, with an average of 131 (120–150) for *S. mussuranae*. Additionally, the distance from the anterior end to the excretory pore is greater in the new species (ave. 279 [191–325]) compared to *S. mussuranae* (ave. 168 [160–185]). Notable differences also exist in buccal cavity dimensions, as *S. orientalis* sp. nov. has a greater width (ave. 23 [14–30] vs 14 [13–15]) and depth (ave. 17 [11–24] vs 11 [8–12]). Furthermore, *S. orientalis* sp. nov. infects multiple organs, contrasting with *S. mussuranae*, which infects only the lung. In terms of geographical distribution, *S. mussuranae* is found in the Neotropical region. Finally, regarding host specificity, *S. mussuranae* infects the mussurana, *Clelia clelia* within the Colubridae family (Kuzmin *et al*., 2019).

### Variation

**Figure 4.**
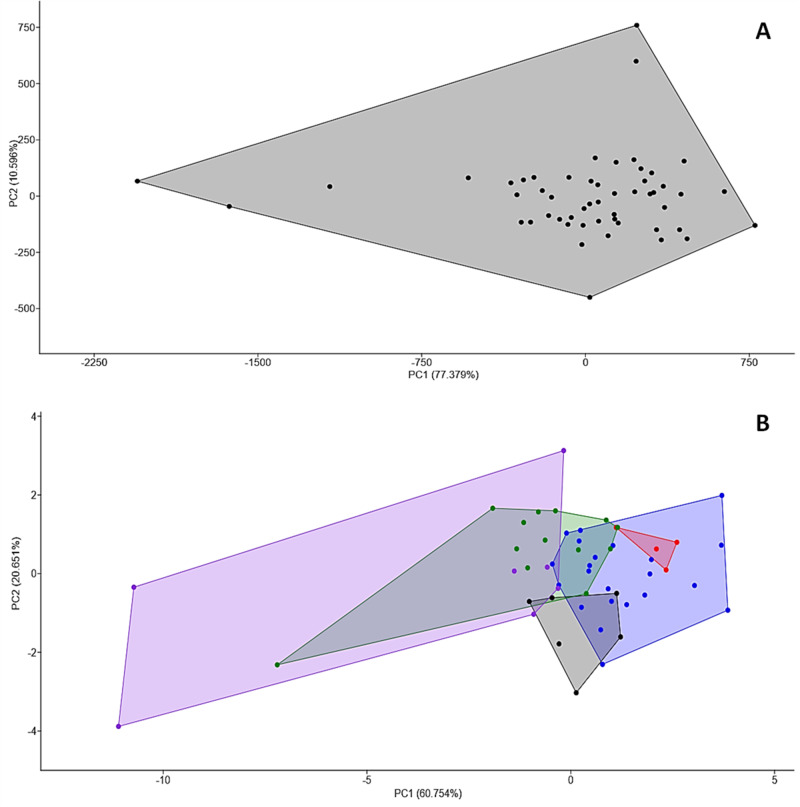
Principal component analysis of *Serpentirhabdias orientalis* sp. nov. specimens: (A) Principal component analysis of all female samples based on 19 examined morphological characters (87.975% of total variance). Black dots and lines represent individual specimens and their group; (B) Principal component analysis of all female specimens based on 23 examined morphological characters (81.405% of total variance). Coloured dots represent individual female *S. orientalis* sp. nov. specimens hosted in different snake specimens, and corresponding-coloured lines reveal their clustering.

To assess the homogeneity of *S. orientalis* sp. nov. without population or strain diversification, morphological measurements of female specimens were analysed. The results indicated no significant morphological clustering among individuals, as demonstrated by a two-dimensional plot of the PCA of PC1 and PC2, which revealed no clear separation (Figure 4A). This absence of differentiation is further supported by PCA accounting for 87.975% of the total variance, with a marked decline in eigenvalues from PC1 (77.379%) to PC2 (10.596%), and from PC2 to PC3 (3.253%).

The intensity of *S. orientalis* sp. nov. infections ranged from 6 to 216 parasites, with a mean of 58. PCA analysis of all female specimens revealed 5 overlapping clusters, accounting for 81.405% of the total variance, indicating some degree of morphological variation among *Serpentirhabdias* specimens from different snake hosts (Figure 4B). Further analysis identified body length as the primary measurement contributing to the observed divergence, particularly among hosts represented by the green and purple clusters (Figure 4B). These findings suggest that body length may be a key morphological feature influenced by host factors, underscoring the need for further investigation into the host-related mechanisms driving this variation.

### Genetic characterization and phylogenetic position

**Figure 5.**
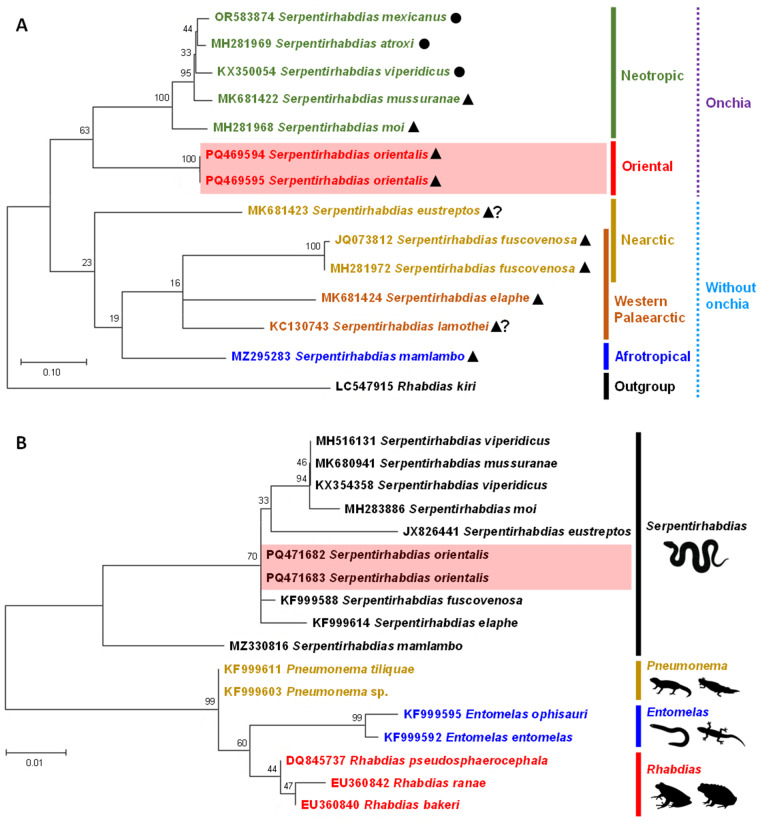
Phylogenetic analysis of available lungworm species sequences from the genus *Serpentirhabdias* and the Rhabdiasidae outgroup, using *COI* (A) and 28S rRNA (B) genes. These analyses were performed with the maximum likelihood method in MEGAX, with branch length scale bars representing the number of substitutions per site. (A): coloured characteristics represent variations in rhabditid helminths sourced from GenBank. *Serpentirhabdias orientalis* sp. nov., found in the oriental region in this study, is highlighted in red (font/line), while other coloured fonts/lines represent species from various biogeographic regions. Dashed lines indicate species with or without onchia. Symbols represent the shape of the oral opening: round (⚫), triangular (▲), and no data/suspected triangular (▲**?**). (B): coloured fonts/lines represent different genera of helminths and the specificity of the infected host groups.

Both phylogenetic analyses (*COI* and 28S rRNA genes) confirmed the genus *Serpentirhabdias* as a monophyletic group (Figures 4A–B), with only the *COI* analysis recognizing *S. orientalis* sp. nov. as a distinct species within the genus (Figure 5A). In the *COI* phylogeny, the species of *Serpentirhabdias*, whether characterized by the presence or absence of onchia, clustered in accordance with their distribution and other morphological traits. Within the group possessing onchia, *S. orientalis* sp. nov. (in the Oriental region) exhibited a close phylogenetic relationship with *S. mussuranae* and *S. moi* (in the Neotropical region), both of which share a triangular oral opening. In contrast, *S. mexicanus* was closely related to *S. viperidicus* and *S. atroxi* (also in Neotropics), which possess a round oral opening. The 28S rRNA phylogeny displayed low genetic variation, making it difficult to clearly distinguish *S. orientalis* sp. nov. However, it confirmed that the genus *Serpentirhabdias* is distinct from other genera of reptilian/amphibian lungworms. All species within the genus *Serpentirhabdias* are associated with snake hosts, while the genera *Rhabdias, Entomelas* and *Pneumonema* infect frogs and toads, legless lizards, as well as skinks and salamanders. This finding also indicates that Neotropical species are separated from those in other regions (Figure 5B).

The genetic distance matrix revealed a clear distinction between *S. orientalis* sp. nov. and its congeners, with genetic divergence for the *COI* gene ranging from 12% to 14% and for the 28S rRNA gene from 0.2% to 4%. Additionally, the genetic sequence variation in the *COI* and 28S rRNA genes between the 2 specimens of *S. orientalis* sp. nov. was 0%, indicating complete genetic similarity.

### Natural history

*Serpentirhabdias orientalis* sp. nov. exhibits a high prevalence in its host, the monocled cobra (*Naja kaouthia*), with all 5 examined specimens testing positive for infection. This species is predominantly located in the lung, but it is occasionally found in the trachea and heart of the snakes, which were captured in Bangkok and surrounding areas. This finding is particularly significant as it represents the first identification of *Serpentirhabdias* sp. in the Oriental region and within the Elapid family.

Currently, only adult female parasites have been documented in the host, with no investigation into lesions or other life cycle aspects at this stage. However, the presence of numerous eggs in the intestinal contents of the host suggests that larvae may hatch externally.

## Discussion

A new species of snake lungworm, *Serpentirhabdias orientalis* sp. nov., was discovered in the respiratory organs of the monocled cobra (*Naja kaouthia*) in Thailand, marking the first identification of a lungworm in an Elapid snake. This worm species belongs to the genus *Serpentirhabdias*, within the family Rhabdiasidae, known for infecting carnivorous reptiles (Tkach *et al*., 2019). Detailed taxonomic analyses revealed that the specimens exhibit distinct geographical, host-specific, morphological, and genetic characteristics that differentiate them from all previously described species within the genus *Serpentirhabdias*.

Parasite species in the genus *Serpentirhabdias* are known for their strong host specificity (Table S1), presumably co-evolving with their reptilian hosts over time. Phylogenetic analyses in this study supports this notion, revealing that *S. orientalis* sp. nov. in Southeast Asia has a deep evolutionary relationship with its host. This long-standing evolutionary history likely reflects the specialized nature of the host–parasite relationship, further indicating a co-evolutionary dynamic shaped by these interactions (e.g. Kuzmin *et al*., 2007; Buckingham and Ashby, 2022). Comparisons have shown that different *Serpentirhabdias* species tend to infect specific snake families (Table S1), highlighting the close link between parasite evolution and host diversification. However, 2 exceptions exist: *Serpentirhabdias fuscovenosa*, reported to infect species from 3 snake families – Colubridae, Boidae and Viperidae (Martínez-Salazar and León-Règagnon, 2006; Tkach *et al.*, 2019; Morais *et al.*, 2017; Kuzmin and Tkach, 2024) – and *Serpentirhabdias eustreptos*, which parasitises both Viperidae and Colubridae (MacCallum, 1916; Kuzmin and Tkach, 2024). The discovery of *S. orientalis* sp. nov. suggests that Southeast Asia may harbour additional, yet undiscovered, parasite species with similarly specialized host associations.

In terms of geographical distribution, the genus *Serpentirhabdias* comprises 22 species found globally (Table S1). In Asia, 7 species have been reported, including *Serpentirhabdias fuscovenosa* (Railliet, 1899; Goodey, 1924), in the Holarctic region; in the Western Palaearctic region, *Serpentirhabdias elaphe* (Sharpilo, 1976) and *Serpentirhabdias martinoi* (Kurochkin and Gus’kov, 1963); in the Eastern Palaearctic region, *Serpentirhabdias horigutii* (Yamaguti, 1943), *Serpentirhabdias agkistrodonis* (Sharpilo, 1976) and *Serpentirhabdias vibakari* (Kuzmin, 1996); and in the Eastern Palaearctic islands, *Serpentirhabdias kurilensis* (Sharpilo, 1976). Prior to this study, no species from this genus had been reported in the Oriental region or Southeast Asia, highlighting the lack of research on lungworms in these areas. Given that Southeast Asia, and Thailand in particular, is home to approximately 250 species of snakes that inhabit a variety of ecosystems (Cox *et al*., 2012; Ratnarathorn *et al*., 2024), it is plausible that other, as yet undiscovered, species of lungworms may exist in this region. Further investigations into other snake species in the same and neighbouring habitats may reveal additional infections by *S. orientalis* sp. nov. or other related helminth species. Continued exploration is warranted.

*Serpentirhabdias orientalis* sp. nov. represents the eighth species of this genus reported in Asia, and the first discovery in the Oriental region. The morphological characteristics of this new species, particularly the structure of the onchia, along with phylogenetic analysis, suggest an evolutionary relationship that is more closely aligned with species found in the Neotropical region (Kuzmin *et al*., 2016, 2021; Morais *et al*., 2017; Machado *et al*., 2018; Velázquez-Brito *et al*., 2024). The morphological and molecular (*COI*) evidence supporting a close relationship between *S. orientalis* sp. nov. and Neotropical species presents a puzzling biogeographical scenario regarding how the parasites dispersed between these 2 continents.

Given that these parasites are highly host-dependent (e.g. Barrett *et al*., 2008; Tkach *et al*., 2019), their evolutionary history differs from that of their snake hosts. Snakes from South America and Southeast Asia share a common ancestor that dispersed across these continents around 100–90 million years ago, during the breakup of Gondwana, particularly in the case of boas and pythons (Pyron *et al*., 2013). Notably, there is no direct evidence of any snake species crossing between Southeast Asia and South America, or vice versa, following the continental isolation after the breakup of Gondwana (Scotese, 2004; Vidal and Hedges, 2009; Pyron *et al*., 2013). The presence of lungworms with close evolutionary ties across these 2 continents suggests that the parasites may have evolved after the diversification of snakes and/or potentially dispersed via other means such as host co-transport (Chaisiri *et al.*, 2019; Cowie *et al.*, 2022). Besides, morphological and genetic similarities observed in these lungworms might be attributed to convergent evolution, a process where unrelated organisms independently evolve the similarities due to analogous selective pressures in similar environments (Losos, 2011). These aspects remain unexplored and represents an intriguing area for future research. Investigating the evolutionary timeline of these parasites in relation to their hosts’ distribution could shed light on the broader biogeographical patterns at play.

Despite the monophyly of the genus *Serpentirhabdias*, the positioning of *S. orientalis* sp. nov. differs between the *COI* and 28S rRNA phylogenies The *COI* analysis indicated a close relationship with Neotropical lungworms, while the 28S rRNA analysis did not provide a clear distinction. This discrepancy may be attributed to the significantly lower genetic divergence observed in the 28S rRNA compared to the *COI*, suggesting that the *COI* gene is more effective for species identification (e.g. Kuzmin *et al*., 2019; Velázquez-Brito *et al*., 2024), whereas the 28S rRNA appears to be limited to genus-level differentiation. Future research focusing on genetics of other species within *Serpentirhabdias*, as well as the examination of additional genetic markers, may yield greater insights into the relationships among these parasites.

The male morphology of *S. orientalis* sp. nov. requires further investigation to enhance species identification. Future studies should explore sexual dimorphism and aspects of the nematode’s life cycle to provide a more comprehensive understanding of this species. The PCA of female specimens revealed distinct clusters, suggesting that individual host specimens may influence the morphological traits of the parasite. In particular, body length was identified as the primary parameter contributing to the observed divergence, likely reflecting differences in the maturity of the nematodes. However, the small sample size from each host highlights the necessity for larger studies to better assess variability related to host factors (Charoennitiwat *et al*., 2023, 2024a, 2024b).

Ecologically, the presence of *S. orientalis* sp. nov. in the cobras prompts questions about the potential impact of parasitic infections on snake populations. Although the observed worm burden of up to 216 individuals per host may not necessarily indicate a high-intensity infection, the effect on host fitness remains uncertain. Given that monocled cobras are commonly found in rural and urban settings (Ratnarathorn *et al*., 2024), further studies are needed to assess the ecological and conservation implications of this parasitism, particularly in stressed habitats.

From a veterinary and ecological standpoint, the discovery of *S. orientalis* sp. nov. underscores the importance of monitoring parasitic infections in reptiles. Although there is no evidence suggesting zoonotic potential, increasing wildlife–human interactions call for vigilance in understanding parasite dynamics. Additionally, parasitic infections in captive reptiles present veterinary challenges, emphasizing the need for biosecurity measures in the reptile trade, exotic pet care and conservation efforts (e.g. Charoennitiwat *et al*., 2024b).

In conclusion, this study reports the discovery and description of *S. orientalis* sp. nov., a new species of lungworm found in the respiratory organs of the monocled cobra (*Naja kaouthia*) in Thailand. This marks the first record of a *Serpentirhabdias* species in Southeast Asia and in an elapid snake. Detailed morphological and molecular analyses confirmed the distinctiveness of *S. orientalis* based on traits such as body size, buccal cavity dimensions and reproductive characteristics, differentiating it from closely related species. The phylogenetic analysis showed a close relationship with species found in the Neotropical region, raising questions about the biogeographical patterns of parasite dispersal. The high prevalence of infection in the cobras and its potential ecological and conservation impacts were noted.

## Supporting information

Charoennitiwat et al. supplementary material 1 Charoennitiwat et al. supplementary material

Charoennitiwat et al. supplementary material 2 Charoennitiwat et al. supplementary material

## Data Availability

The data that support the findings of this study are available from the first and corresponding authors upon reasonable request.

## Supplementary materials

The supplementary material for this article can be found at https://doi.org/10.1017/S0031182025000174.
