# *Serpentirhabdias orientalis* sp. nov. (Nematoda: Rhabdiasidae), a new lungworm species in *Naja kaouthia* from Thailand: the first record of the genus from the oriental region and an elapid snake – CORRIGENDUM

**DOI:** 10.1017/S0031182025101339

**Published:** 2026-02

**Authors:** Vachirapong Charoennitiwat, Supakit Tongpon, Phatthariya Suksuwan, Kittipong Chaisiri, Panithi Laoungbua, Tanapong Tawan, Urusa Thaenkham, Napat Ratnarathorn

The corresponding author, Urusa Thaenkham, was omitted from the original article. The corresponding authors for this article are Urusa Thaenkham and Napat Ratnarathorn.

The article has been updated.

**Results**

(On page 242 of the paper), please add: **ZooBank LSID:** urn:lsid:zoobank.org:pub:53C13869-D15C-4AEC-BD47-228067E94B0D

(On page 241 of the paper, Paragraph 4^th^), please change from “55 cycles” to “35 cycles”: ...For the 28S rRNA primers, the profile began with an initial denaturation at 94 ∘C for 3 min, followed by **35 cycles** of 94 ∘C for 30 sec, 54 ∘C for 30 sec and 72 ∘C for 1 min, with a final extension at 72 ∘C for 7 min (Dare et al., 2008)....
